# Supplementary material for: KEAP1-NRF2 Interaction in Cancer: Competitive Interactors and Their Role in Carcinogenesis
Source: Cancers (Basel). 2025 Jan 28;17(3):447. doi: 10.3390/cancers17030447 (PMC11816071; doi:10.3390/cancers17030447)
Supplement: Supplementary file 1 [file cancers-17-00447-s001.zip › cancers-3417262-supplementary.pdf]

Supplemetary information

The exact three-dimensional structure of the DPP III–Kelch complex has not yet been experimentally determined. However, several combined experimental and computational studies have been conducted to provide insights into the structure of the complex (in both of which members from our laboratory participated):

> M. Gundić, A. Tomić, R. C. Wade, M. Matovina, Z. Karačić, S. Kazazić, S. Tomić; Human DPP III–Keap1 Interactions: A Combined Experimental and Computational Study, Croatica Chemica Acta 89(2) (2016): 217–228.
>
> S. Matić, I. Kekez, M. Tomin, F. Bogár, F. Šupljika, S. Kazazić, M. Hanić, S. Jha, H. Brkić, B. Bourgeois, T. Madl, K. Gruber, P. Macheroux, D. Matković-Čalogović, M. Matovina, S. Tomić; Binding of Dipeptidyl Peptidase III to the Oxidative Stress Cell Sensor Kelch-like ECH-associated Protein 1 is a Two-step Process, Journal of Biomolecular Structure and Dynamics, 39(18) (2020): 6870–6881.

In the latter study, Small-Angle X-ray Scattering (SAXS) analysis and Hydrogen/Deuterium Exchange (HDX) mass spectrometry experiments provided insights into the overall shape of the complex and the regions of the proteins involved in the interactions, respectively. MD simulations of the complex in both studies were performed on the scale of several hundred nanoseconds.

With advancements in computational technology enabling MD simulations on increasingly longer timescales, as well as computational tools that facilitate protein-protein structure predictions (e.g., AlphaFold-Multimer and AlphaFold3), we extended our computational study on this subject, aiming to determine the most reliable complex structure. This was achieved by combining longer, microsecond-scale MD simulations with protein-protein structure prediction results. More specifically, several structures of the DPP III–Kelch complex were built based on our previous findings and AlphaFold predictions and subjected to numerous microsecond-long MD simulations using program Amber 22. Final trajectories were analyzed via clustering to identify the most populated complex structure, which is assumed to represent the most energetically favorable conformation. This structure is depicted in Figure 3.
